# Police Body-Worn Cameras as a Response to Domestic and Family Violence: Practitioner Insights Into the Consequences for Victim/Survivors

**DOI:** 10.1177/10778012231185541

**Published:** 2023-07-24

**Authors:** Mary Iliadis, Bridget Harris, Zarina Vakhitova, Asher Flynn, Danielle Tyson

**Affiliations:** 1 School of Humanities and Social Sciences, Deakin University, Melbourne, Victoria, Australia; 2 2541 Monash University, Melbourne, Victoria, Australia

**Keywords:** body-worn camera technology, police, domestic and family violence, misidentification, ideal victim

## Abstract

Body-worn cameras (BWCs) have been promoted internationally to enhance responses to domestic and family violence (DFV). However, little is known about their utility, benefits, and limitations. Drawing upon the insights of DFV practitioners who support victim/survivors in the Australian states of Queensland and Western Australia, this article finds that while BWCs can capture some DFV incidents, they are unable to show their full context and impacts. BWC footage may also have consequences for “nonideal” victim/survivors, including wrongful criminalization and the removal of children. Ultimately, we argue that trauma-informed responses are vital for BWC use in DFV cases to improve frontline responses.

## Introduction

Domestic and family violence (DFV) is a serious social and public health issue worldwide, with the World Health Organization (WHO) estimating that one in three women have experienced DFV in their lifetime (Meyer & Frost, 2019; Stubbs & Szoeke, 2021; WHO, 2021). DFV overwhelmingly, although not exclusively, affects women and children. In Australia, the Personal Safety Survey estimates that one in six women have experienced physical or sexual violence by a current or previous intimate partner since the age of 15 (Australian Bureau of Statistics, 2017). DFV has extensive health, social and economic consequences for victim/survivors and communities. The annual cost of DFV in Australia is estimated to be in excess of $AUD22 billion (KPMG, 2016), and it is one of the leading causes of death, illness, and disability for women under the age of 45 (Australian Institute of Health and Welfare [AIHW], 2021). Other consequences include emotional, psychological, physical and/or financial harm, homelessness, loss of income/employment, removal of children, and drug and alcohol addiction (Donovan et al., 2016). Globally, the public and private costs of violence against women are estimated at $1.5 trillion (USD, see UN Women, 2016).

The prevalence and consequences of DFV ensure it is a policing priority, and this has led to several justice system interventions, innovations, and reforms (Islam & Mazerolle, 2022). One such reform is the use of police body-worn camera (BWC) technology, which has been introduced across parts of the UK, the USA, and now, Australia.

BWC technology has been touted as a “game changer” due to its potential to reduce victim/survivor court appearances and alleviate the harm, discomfort, and danger associated with formal responses (Cardozo 2015). It has also been identified as having the potential to strengthen evidential cases (Goodall 2007; Owens et al. 2014), improve the probability of guilty outcomes (Morrow et al. 2016), lessen resource expenditure (Braga et al. 2017), and bolster public perceptions of transparency, accountability, and fairness in police decision-making processes (Harvard Law Review 2015; Iliadis et al., 2022). However, there are potentially negative consequences that can arise. Given the incident-based and visual focus of the medium, critics question whether BWCs can fully capture DFV, particularly nonphysical harms or coercive behaviors. There is also a risk that effects of violence (such as trauma) might be misunderstood and/or misidentified, resulting in the criminalization of victim/survivors, and potential for perjury charges if there is a substantive deviation from recorded footage in court statements (Harris, 2020). Footage might also be used to pressure victim/survivors to proceed with matters, which ignores their agency and reasons for reluctance, including that violence and risk may escalate. Privacy concerns have also been flagged on the recording and storage of, and access to, data (Harris, 2020).

As gatekeepers of the legal system, and often first responders to DFV incidents and victim/survivor disclosures, it is vital to understand the impacts of the use of BWCs by police in DFV contexts. Some research has sought to do this (Cubitt et al., 2017; Iliadis et al., 2022; Islam & Mazerolle, 2022; McCulloch et al., 2020) by evaluating BWC trials through police perceptions, interviews with court officials and law enforcement staff, or with victim advocates. Few studies have engaged victim/survivors (with the exception of Barlow, 2022; Harris, 2020; Saulnier et al., 2022; Simpson, 2021). However, there is still much to be explored on the effects, benefits, and risks of BWC use in DFV incidents and proceedings, particularly through the vital, practice-based knowledge of frontline, and DFV support services.

This article responds to this knowledge gap, drawing on a multi-jurisdiction qualitative study of BWC technology in the policing of DFV in Australia. Specifically, this article presents findings from 30 in-depth interviews with DFV stakeholders in 2 Australian jurisdictions where BWC technology has been implemented in DFV policing: Queensland (QLD) and Western Australia (WA). The article aims to shed light on DFV practitioner perceptions and experiences of the use of police BWCs in DFV responses, including its potential benefits and risks. In presenting these insights, we draw on the critical knowledge that is held by those who work directly with clients experiencing DFV within the support services sectors, including DFV, sexual assault, legal and court services, behavior change programs, and specialist diversity services.

The article begins by providing a brief overview of BWC deployment in Australia and internationally, drawing on key emerging literature. We then present the study methodology, followed by a discussion of key findings on the potentials and consequences of BWC use in DFV contexts. Specifically, we argue that while this technology has the potential to impact positively on policing practice and reduce the trauma of secondary victimization associated with victim/survivor testimony, we agree with Simpson (2021) that they are not a “panacea” for poor policing or for “solving” DFV (p. 2). Indeed, the way in which BWC footage is viewed and interpreted can lead to the unnecessary criminalization of victim/survivors, for example, where they capture footage of victim/survivors who are distraught or inebriated, and in some cases, it can call into question victim/survivor credibility (Harris, 2020). This is particularly so for victim/survivors who do not conform to “ideal victim” constructions (Christie, 1986; Meyer, 2016; Wheildon et al., 2022), and this may have consequences for the removal of children. Our findings further point to the importance of appropriate training and upskilling of police and other criminal justice agents (including relevant court staff) in using and interpreting BWC technology. We conclude by outlining the study limitations and need for further research, in particular, for work that engages with victim/survivors.

### BWC Deployment in Australia and Abroad

BWCs are a staple in many police organizations. Generally, the technology is deployed in callouts as opposed to being used for specific crime types or units. There have, however, been a series of DFV applications and trials, including in Arizona (US, see Katz et al., 2014; Morrow et al., 2016), Plymouth (UK, see Goodall, 2007), Wolverhampton (UK, see Drover & Barak, 2015), and Essex (UK, see Owens et al., 2014). There is a growing body of literature exploring the utility and impacts of BWCs (Ariel et al. 2015; Clare et al. 2021; Gaub et al. 2021; Lum et al. 2019; White & Malm 2021). BWC trials have been undertaken by police agencies across the US, the UK, Canada, and Australia, however, an empirical review by Lum et al. (2019) suggests that research on BWCs is mostly US-based and 32 (out of 70) studies are focused on the impact of BWCs on police officers’ behaviors, and police attitudes toward BWCs. Research on police views and attitudes toward BWCs have found a general consensus that police support BWC use and outcomes (Gaub et al. 2018). For instance, Lum et al. (2019) found that police believed BWCs would protect officers from the public, including from vexatious complaints, and enhance police behavior and performance. Research by Gaub et al. (2018) in the US found that police felt BWCs would strengthen evidence collection and quality, thereby supporting prosecutions.

Results on BWC effectiveness in responding to DFV and assisting victim/survivors are mixed, and it is worth noting that success is largely associated with the progression of cases through the legal system. Some research has found that BWC footage increased the likelihood of cases being initiated and resulting in a conviction (Katz et al., 2014; Morrow et al., 2016) or evidentiary quality and sanctions (Goodall, 2007). Other research has been less assured, reporting no discernible impact on improved evidentiary impact (Drover & Barak, 2015; Owens et al., 2014). Some scholarship has considered how BWC footage impacts juror decision-making and points to a need for more insight into how prejudices and biases impact viewing and responses in judicial spaces (Smith et al., 2019). An enquiry from New Zealand suggested that, in contrast to written statements, video statements increased the likelihood of obtaining a guilty plea by 77% (Walton et al., 2018). In the US context, a study by Backes et al., (2021) suggested that video-recorded statements can offer further information about the incident, scene and DFV victim/survivor, aid prosecutorial negotiations with the defense, assist prosecutorial trial strategies (pertaining to evidence and jury management), and bolster DFV engagement in cases.

In the Australian state of New South Wales (NSW), DFV-specific BWC applications were first utilized in 2015 to allow for the admissibility of pre-recorded evidence either in-chief, in whole, or in part (Procopis, 2018). Deployment and pilots of BWCs in DFV incidents were then implemented in the state of Victoria (McCulloch et al., 2020). In QLD, the uptake of BWCs in DFV responses occurred following the 2015 Special Taskforce on DFV (QLD Government, 2015). Legislative and operational shifts have also enabled the rollout of BWCs in DFV policing responses in other Australian state and territory jurisdictions, including the Northern Territory (in 2017, after 2016 pilots; Banks, 2016) and Tasmania (trialed in 2018, and expanded in 2019; Tasmania Police, 2018). The state of WA trialed BWCs in 2016 for 6 months and then disseminated the technology to all frontline police in mid-2019. By July 2020, the WA Police Force completed its BWC rollout to frontline police, including to the Tactical Response Group; at the time of writing, this is the only police agency in Australia that has done so (Clare et al., 2019, 2021).

A 2015 and 2016 internal review of the NSW Police initiative was not released, but a 2017 evaluation found BWCs had no observable impact on guilty outcomes (Yeong & Poynton, 2017). An assessment by camera manufacturer, Axon (2017), claimed QLD Police BWC evidence “reduced police summary court hearings by 60%” (p. 1); while a study featuring Victorian stakeholders (police, court personnel, legal practitioner, and DFV sector workers) reported a consensus that BWCs would enhance DFV responses and outcomes for victim/survivors (McCulloch et al., 2020). An Australian qualitative project, drawing on interviews with DFV victim/survivors and law enforcement, found that police believed the technology used in NSW was effective in reducing complainant time and police and prosecutorial resources, and was seen as a “truer” account of the event (Simpson, 2021, p. 244). Contrasting Yeong and Poynton's (2017) early study, police participants believed that BWCs increased prosecutions and felt there was “contrition or acceptance in perpetrators for what they have done” when the footage was viewed (Simpson, 2021, p. 245). The composure of victim/survivors and technical issues, however, were said to affect evidentiary quality.

Most of the aforementioned studies engaged police. However, there remains a significant knowledge deficit capturing the views of other stakeholders, such as those working with the DFV support sector and victim/survivors, underscoring the importance of accounting for their perspectives and experiences alongside police.

## Research Design

The current BWC project is a mixed methods study, including a survey with Australian police members (*n *= 452), in-depth interviews with DFV stakeholders (*n *= 30), and victim/survivors (*n *= 15). Ethics approval was received from the Deakin University Human Research Ethics Committee (DUHREC) in 2019 (approval number: 2019-297). This article presents findings specifically from the in-depth interviews with frontline and support service providers.

Between May and September 2020, 30 DFV stakeholders were interviewed on the Zoom platform across QLD (*n *= 24) and WA (*n *= 6).^1^ Participants were recruited via the professional networks of the team, including sending emails to relevant organizations, advertisements through stakeholder newsletters and bulletins, and social media. The participants had a variety of seniority and experience, including CEOs; solicitors of women's specialist and legal services; individuals working within DFV integrated and coordinated response teams; high-risk systems’ advocates and coordinators; women's advocates situated within men's DFV intervention and education programs; counseling and crisis practitioners; DFV case workers located within police stations; policy, development, and coordination advisors; family counselors; and women's DFV court advocates.

Participants were asked a series of questions on three key themes: (a) the usefulness of BWCs in policing DFV, including for evidentiary purposes; (b) the potential benefits of the use of BWCs in DFV responses, including for victim/survivors; (c) the potential risks of BWC technology, especially in relation to its adverse consequences for victim/survivors. The interviews embodied a framework that was sensitive to the issues being addressed. They lasted approximately 1 h and were conducted one-on-one or in a group setting. With permission, the interviews were recorded and transcribed by an external transcription company, subject to confidentiality requirements. Participants were assigned pseudonyms containing the letter P and number, as well as a brief but purposeful description of their role, such as P1 (team manager, violence prevention reform, WA).

The de-identified interview transcripts were uploaded into NVivo (coding software platform) for thematic analysis. We conducted line-by-line coding of each transcript, and key themes were identified according to the frequency with which they appeared across the transcripts, as calculated by NVivo. Each transcript was coded at least twice and by at least two researchers. The coding tool enabled the team to discern and compare key trends across the data (Nowell et al., 2017). Overall, we identified 40 thematic codes, which were then amalgamated into overarching themes. For the purpose of this article, we focus on themes related to: whether, and to what extent, BWCs capture DFV and its harms; the effects of ongoing abuse and trauma; benefits and consequences of BWCs; ideal victim/survivor constructs; and BWCS and the removal of children.

## Results

The findings presented in this article are grouped within overarching themes of the potential utility, benefits, and risks of BWC use in DFV responses. First, we present data which speaks to the capacity for BWCs to capture DFV and its impacts, including their ability to provide real-time, irrefutable evidence. In this section, we also address participants’ concerns about the risks associated with the capturing of footage, insofar as it is incident specific and generally only captures evidence of physical harms, meaning that other harms and impacts can be omitted. Second, we outline the risks and impacts associated with capturing, interpreting, and misunderstanding the effects of victim/survivor trauma. Third, we caution that without sufficient knowledge and understanding of DFV and its manifestations, victim/survivors who deviate from “ideal” victim constructs (Christie, 1986) might be especially prone to adverse consequences arising from the use of BWC footage. Finally, we highlight participants’ concerns around how these issues can culminate in the removal of children.

### BWCs Providing Real-Time Footage and Tangible Evidence

While there is an extensive body of research on BWC use in generalist responses (see, e.g., Cubitt et al., 2017; Lum et al., 2019), the same cannot be said about BWC use in DFV-specific applications. Although, the few studies that have been conducted suggest that BWC footage promises to offer an objective account of an incident, the accused's behavior and its impacts on victim/survivors, including any damage to property, children, or bystanders (Harris, 2020; Iliadis et al., 2022). Some participants in our research maintained that BWCs can achieve these promises. As noted by P24 (coordinated response service worker, police, and child protection, WA), BWCs “can allow for accurate data to be captured [such as] the dynamics in the house, … which would be really beneficial.” Another participant similarly explained how BWCs can document “the scene, the severity, … the damage around the house, children distressed, the woman distressed; that can be hugely useful in a court” (P11, CEO domestic violence support, QLD). P18 (administrative and family violence practitioner, QLD), likewise contended that BWCs show:

Some of the impacts of the violence, so particularly around documenting property damage, and … if there are visible injuries following an assault or an incident in that, hopefully capturing them better than a flat photo is able to. It may be able to capture the significant impact on a woman … [and] depending on the perpetrator's behavior, how aggressive he is during an incident, conveying to the court how fearful it would be to be in the presence of somebody using that type of violence.

Some participants also described how the evidence resulting from BWC footage can reveal the impact of harm on children. As P27 (team leader, coordinated response DFV, QLD), explained:

If there's recordings of … children screaming and crying, yes, you’re visually seeing those impacts. And that's a lot different to reading a police statement or a police's perspective on things, for someone to actually be seeing and hearing what's actually happening can be a lot more realistic.

Another participant similarly reflected on this possible benefit, providing an example of how it assisted a victim/survivor in court proceedings:

the police attend[ed] the domestic violence incident and the application was lodged on the aggrieved, on the mum's behalf, and two of the children were present …, but then the aggrieved contacted the … attending officer, and asked them if they can request the children to be made under the protection. The original officer said no … but then the sergeant … actually went back on the camera and then looked at it. They [the officers] did actually interview the children and the children said that they are terrified of their dad and they don’t want to have contact with their dad. So the children's names were then being added. (P13, DFV court advocate, QLD)

It was therefore evident that many participants felt that BWCs could document the children's distress, which may have been lost had the decision been based solely on the police's written account or recollection. The potential for BWC footage to work effectively in such scenarios was a common theme both for ensuring a more accurate description of events, but also for police recall. As P27 (team leader, coordinated response DFV, QLD) explained:

Often things can happen very quickly. You [the police] are at a scene and things can go down there, and then, there's lots said. It's very hard to pick up on everything, particularly if say, you [the police] are single crewed, and you are having to have conversations with both parties, or try and separate the parties. And so, you might be able to then go through that footage to say “oh, what did that person say again?” and clarify things.

The notion that BWCs can capture a sense of “reality” of DFV and its harms was also positively noted some by participants. P28 (senior solicitor, women's legal service, WA) maintained that BWCs “can be very, very powerful in detailing or sort of evoking for the watcher, the nature of the violence.” She continued, “there's an upturned table, there's a hole in that wall, the house is trashed—it can be … powerful in that instance.” P18 (administrative and family violence practitioner, QLD), similarly claimed:

It would shine a light on what the realities of domestic violence is. … It's got more severe, the type of violence women are disclosing, there is a lot more sexual violence being disclosed, there's a lot more strangulation. …The community doesn’t necessarily hear those stories because they’re not published or reported on in the media, and so they don’t necessarily know the seriousness of it.

In this way, BWCs can be of benefit to some victim/survivors, the police, and the community, offering an educative and possibly preventative function, although it should be noted that not all communities may view BWC technology in this way because of its association with and deployment by the State. This is particularly so for First Nations women and other communities of color who have experienced hyper-criminalization and mass incarceration, and who have reported negative experiences with State agents, justice officials and interventions (Goodmark, 2021).

Participants also pointed to BWCs as providing an opportunity to assist victim/survivors in the court process, for example, “in not having to provide further additional evidence … and reduce the burden on the women to provide that evidence” (P18, administrative and family violence practitioner, QLD). In this sense, as P24 (coordinated response, police, and child protection, WA) explained, BWCs might alleviate the burden associated with giving witness statements and testifying:

If the victim's really traumatized and it's right after the incident, they might have said something, or forgot, or timeframes might be misjudged and that sort of thing, so it would be helpful in that way. It would give them a more accurate account of what happened straight after.

P11 (CEO, domestic violence support, QLD), similarly commented:

We know from women that they don’t talk because they don’t feel safe to talk, and because their experience of the criminal justice system is they’re not believed. … To actually have the visual and sound recordings to how he presents … might actually provide veracity or credibility to what she [the victim/survivor] is saying.

This could reduce some of the secondary trauma of the court process (Iliadis, 2020), and offer meaningful ways for victim/survivors to have their experiences seen and heard. It may also ensure courts address situations in which perpetrators deny or downplay the severity of their actions, or falsely label the victim/survivor as the primary aggressor.

### BWCs Merely Capturing “Incident-Based” Responses?

While the participants suggested ways in which BWCs can provide an objective, real-time account of an incident, previous research has documented various technical and practical factors, including jurisdictional differences in BWC applications and procedures, which can influence how BWC footage is viewed and interpreted (Harris, 2020). Furthermore, most participants—even when reflecting on some of the potentials of BWCs—noted that it cannot provide a complete picture of the extent and impacts of DFV. As P14 (women's advocate, men's domestic violence intervention and education, QLD) observed, it captures “a single point in time with no context or no preceding information, and it can give a very distorted view of what the woman's reality is and what the actual violence [is] that's happening in the home.” BWC footage documents an isolated incident as opposed to the patterns that characterize DFV. Indeed, this is true of many criminal justice responses and interventions more broadly, which are sparked by individual events.

P28 (senior solicitor, women's legal service, WA), also noted that capturing a video of the home environment might be powerful where there is physical violence and aggression, but less so when “it's just emotional or it's just verbal [abuse].” This is because context (patterns, intentions, behaviors, and impacts) cannot clearly be shown in BWC footage (see also, Harris, 2020; Simpson, 2021). This might further hinder the capacity for victim/survivors to be believed and have their experiences validated, particularly if police lack a “DV lens and that really good knowledge and understanding of the dynamics and the patterns and reactions [of abuse and perpetrators]” (P27, team leader, coordinated response DFV, QLD).

### Understanding and Responding to the Effects of DFV Trauma

Concerns have emerged in medical research about the lack of understanding among criminal justice personnel about the effects of DFV, including its short- and long-term impacts (Epstein & Goodman 2019; Harris, 2020; Haag et al., 2019; Nemeth et al., 2019). Literature has shown that exposure to DFV can impact cognitive and psychological abilities, and neurological outcomes. These impacts are exacerbated for victim/survivors who experience traumatic brain injury (TBI) as a result of physical force to the head, neck, or body—the effects of which can extend to memory lapses, concentration difficulties, reasoning and communication difficulties, and emotional dysregulation (Nemeth et al., 2019). Recent research has found high prevalence rates of TBI among DFV victim/survivors, although it is commonly under-detected and under-reported (Baxter & Hellewell, 2019).

Epstein and Goodman (2019) have identified that the effects of psychological trauma are akin to the impacts of neurological trauma, whereby confusion, amnesia, dissociative states, and “triggers” may result in traumatic episodes (p. 410). Bearing this in mind, victim/survivors may present as unexpectedly hostile, for instance, in BWC footage (Goodmark, 2008). Additionally, they may not offer consistent testimony in BWC footage and court appearances. As Epstein and Goodman (2019, p. 410) explain, to a DFV trauma expert, the “disjointed, incoherent way that she [a victim/survivor] presents makes it all the more plausible.” Yet, if police and judicial officers do not understand the impacts of trauma, “the opposite is true. … Her story's disjointed, inconsistent nature makes it sound *im*plausible” (p. 410). Accordingly, victim/survivors’ narrative in these scenarios might be seen to lack consistency creating a perception that they are “unreliable witnesses” (Harris, 2020; see also Epstein & Goodman, 2019).

These issues were similarly observed by participants in our research. P11 (CEO, domestic violence support, QLD), for example, claimed, “we’ve had instances where women have been concussed at the scene and police have interpreted it as she's under the influence or something, and they’ve walked away without taking any action, just saying she's drunk.” P18 (administrative and family violence practitioner, QLD), reflected on several examples where women have responded to the abuse with violence themselves, and she raised concerns that this could be interpreted in BWC footage as aggression:

It's very common for women who have had assaults committed against them to react in aggressive and violent ways as a response to the trauma. …. Some of my concerns around body-worn cameras is that distress is misinterpreted as aggression, or is misinterpreted as them being the predominant aggressor … and that the body-worn camera evidence without context or without a domestic violence or a gendered violence analysis of it, could be used by a perpetrator against the woman to either get a protection order against them or have criminal charges laid against them, without an understanding of the impact of violence on someone's ability to regulate their emotions.

In reflecting on their experiences, P14 (women's advocate, men's domestic violence intervention and education, QLD) further unpacked the interconnectedness between the issues associated with incident-based responses and misreading trauma:

I’ve worked with a number of women who have significant barriers to communicating their experience, whether that be language barriers or mental health or trauma-based behaviors. In some cases, women who have AOD [alcohol and drug] challenges who might present as intoxicated, they’re not able to communicate their experience of what's happened, so they’re communicating through their behavior, which can be misinterpreted. … I’ve worked with a woman wh[ere] the perpetrator was attempting to sexually assault her. Police were called. She answered the door with no clothes on, but because she was incredibly traumatized and there had been a level of mutual substance use prior to the assault happening, police interpreted how she was presenting as being a result of her intoxication, not as her fear of being sexually assaulted.

Similar concerns were raised by P11 (CEO, domestic violence support, QLD), who commented on how trauma can affect responses and police may consider this to be “unusual”:

What if she's not communicative? What if she's traumatized because she has just been sexually assaulted and she's not hysterical? Unless you’ve got a really good DV lens and know what you’re looking for, and equally, the people who are seeing that in a court … unless they have that lens, how do they know what … they’re looking at?

This type of experience links in with research on victim/survivors of sexual violence more broadly, where a lack of resistance or freezing is taken to mean the rape was consensual (Burgin & Flynn, 2019), despite this being a common trauma/survival response. Training and upskilling police and judicial officers to better understand the nature and impacts of trauma on victim/survivors is thus crucial, as their interpretations of BWC footage might be distorted without this knowledge, leading to deleterious consequences for victim/survivors, including criminalization.

### The Criminalization of “Nonideal” Women

The “ideal victim,” originally coined by Nils Christie (1986), and later reappraised by Strobl (2004), Dignan (2005), and van Wijk (2013), is premised upon the notion that true and legitimate victim status requires that victims ought to have met—or at least mostly adhere to—a set of restrictive attributes at the time of their victimization, including that they were: carrying out a respectable activity; deemed blameless for their victimization; struggling valiantly against an unknown “big bad” attacker; and reported the offense(s) immediately (Christie, 1986; Meyer, 2016). In the context of DFV, Strobl (2004, p. 298) notes that “ideal” victims should “[cooperate] perfectly with the police and the courts.” There are several barriers that might impact a victim/survivor's capacity to conform to this classification. For example, DFV victim/survivors are rarely viewed as “blameless” or “true,” especially if they do not present as expected or if they are reluctant to proceed with a matter. Additionally, not all DFV is reported to police immediately, if at all (Iliadis, 2020; Meyer, 2016). Yet any deviation between victim/survivors’ characteristics and/or behaviors may result in them being seen as unreliable or untruthful, and less support/sympathy afforded by the courts (Epstein & Goodman, 2019).

Accordingly, idealized constructs of victim/survivors of DFV may influence how BWC is viewed and interpreted. A lack of knowledge about the dynamics of DFV could also result in misidentification of victim/survivors as perpetrators in situations where they might use violent resistance or self-defense (Harris, 2020; Hayes, 2013). Our analysis shows what we had earlier proposed; that the effects of this are particularly notable for First Nations women, women of color, pregnant women, women with disabilities, and immigrant women who are disproportionately criminalized, especially in locations with pro- and dual-arrest mandates (see also Harris, 2020). Speaking to this, one participant stated:

[BWCs] generally supports the biases that already exist. … From a socially constructed perspective, good victims … tend to have a better experience of the justice system throughout and it feeds into … victims who are less credible. It really affects Indigenous women and women from [diverse] backgrounds often as well, particularly if they have a white husband. … The big risk of getting it wrong at that [first] point [of contact], is that they carry … that with them throughout their engagement with the system. (P2, manager, government policy, development and coordination, QLD)

P11 (CEO, domestic violence support, QLD), similarly cautioned how BWCs risk capturing isolated incidents, at the expense of lacking a deeper contextual overview of the broader experiences of the abuse and its impacts. She identified this as a particular concern for women from marginalized communities:

If they [the police] don’t locate that incident in the pattern [of abuse], and all that the court's relying on is what's been captured which can show the woman … might be really angry, and … police have shown up, … if they only take that … and not locate it within the pattern of what she's experienced over time or within his pattern with other women, then it [the BWC footage] can be incredibly destructive.

As these comments suggest, this is particularly problematic if BWC footage of a nonideal victim “acting out” may then frame the DFV matter in court. P18 (administrative and family violence practitioner, QLD), likewise reflected on how the consequences of an incident-based response for those who deviate from “ideal victim” constructs can result in misidentification:

[When] a police officer attending that incident doesn’t know that context when they’re arriving, and they just see a hysterical or angry woman … and go, “Ok, well, she's obviously the problem. Let's remove her and criminalize her, charge her.” … without the context of what's actually happened beforehand. … [BWCs] can be beneficial, I suppose, in some circumstances, but I think there is a risk of it being taken out of context, and that's the case of many types of evidence, is that it has to be [interpreted] within the context that it occurred.

Bearing these misidentification issues in mind, awareness of how perpetrators may endeavor to manage their image and deny, minimize, excuse, and justify their behavior while interacting with police and judicial officers is also relevant (Bancroft, 2002). P1 (team manager, violence prevention reform, QLD), highlighted the lengths perpetrators may go to in order to manage their image and create a perception that they are the victim:

We’ve worked with women whose partners have been horrifically violent to them but then the male partner …he's harmed himself, he's damaged his own property and then he's gone and called police, and none of that is visible to a body-worn camera. Only what he says and what she says, and often he's presenting as much more credible and he's presenting as the victim.

In a similar vein, P18 (administrative and family violence practitioner, QLD), noted:

We have men that will go from 100 to zero as soon as the police car pulls up, and will act as if he's chatting to mates down the pub because he is so in control of his behavior, so for offenders like that, it [the BWC] is not going to capture evidence of their behavior.

Such examples indicate why it is important the BWC footage is not considered a complete and entirely accurate depiction of a DFV incident and must be interpreted with caution.

Participants also pointed to examples where victim/survivors’ words may be interpreted inaccurately in BWC footage. For example, P14 (women's advocate, men's domestic violence intervention and education, QLD), noted: “we know statistically that women overestimate their use of retaliatory … violence and men underestimate [it]. So, the incongruences of that being recorded could absolutely be misused.” P15 (women's advocate, QLD), similarly claimed:

When the police turn up, something's happened, and he says, “oh, she pushed me”, and women go, “yeah, I did”, and they admit to it, but he's lying about everything he's done and as a woman she just, the way we are socialized differently, she’ll admit to it. [So] that can be used against her, but he's not admitting to any of the things that [he did] …..

P14 (women's advocate, men's domestic violence intervention and education, QLD) pointed to victim/survivors having a “misplaced compassion” that also contributes to understating their experiences of abuse, legitimizing the perpetrator's behavior and/or overestimating their use of retaliatory violence, all of which could be incorrectly interpreted in BWC footage:

That misplaced compassion that women invariably have … can be then misinterpreted by police and the courts. … It's really tricky because the last thing you want … is the system using all of that to replicate power and control over her. … If she's actively not saying I want an order or I want an arrest, … well, [the consequence could be] … this is what the body-worn camera shows us, we act upon it.

These concerns are vital to consider, given that it can impact how BWC footage is interpreted in police risk assessments and in courts. P13 (DFV court advocate, QLD), recounted her experiences of supporting a victim/survivor through the justice system, specifically where the BWC “was used against her”:

She lost her kids in the court. … She told the police that he recorded an incident of him assaulting her, and then the police officer said “so, what are you trying to do?” and she said, “I just want to get my evidence”. When that evidence came through the courts, [it looked as though] … she was setting him up.

In another example, P11 (CEO, domestic violence support, QLD), recounted an incident of DFV which could be perceived as “mutual combat … but if you go back in the patterns [of violence], he's known to us [the DFV agency] for protection orders for three other women; she's not.” These insights highlight the importance of training and upskilling police and judicial officers in the interpretation of BWC footage, and again, reiterate that it should not be used as standalone evidence.

### Child Removal

Net-widening and criminalization (see (McKay & Lee, 2019) are also potential BWC problems, as what is captured on BWC footage—illegal materials in the home, for instance, or a victim/survivor who is presenting as hysterical/volatile—may result in charges being issued against victim/survivors. In these contexts, BWC footage may also be utilized against victim/survivors to cast doubt on their parenting, which may impact custody arrangements and result in the removal of children. These consequences are heightened and have serious implications for particular communities, such as for First Nations women, for whom a history of State sanctioned victimization through the segregation and removal of children from their families, and the high rates of charges, convictions and wrongful criminalization, has affected how Indigenous women continue to perceive and experience their interactions with State agents (Goodmark, 2021; Harris, 2020; Murphy, 2015).

Some participants explained that BWC deployment could facilitate positive outcomes for victim/survivors in some situations where children's safety and security appears to be compromised:

Using body worn camera footage in that particular area would be really beneficial because it would be able to capture not only the interaction[s], … but the surrounding[s]: the house, the state of the living conditions and the interaction between the children. Like if they were scared, their interaction to the police as well can sometimes be an indication [that there is DFV]. … If children are really unfazed or it seems like quite an usual occurrence, that can be something that would contribute to family court proceedings or child protection issues. … But then also it depends though, because if there's not an open case with child protection, then it can be quite difficult for them to provide support, but I think that it's good because it can capture, everything really in a firsthand account of what's happening. (P24, coordinated response service worker, police and child protection, WA)

On the other hand, P12 (high-risk systems advocate, integrated response, QLD) explained how it could also have negative repercussions for the child being exposed to violence:

If kids are a part of it, then that can work against mum in her being a protective parent or children being exposed to family violence so it can have a negative impact on the parent's [status] with the child.

P11 (CEO, domestic violence support, QLD) highlighted concerns around how BWC footage may be misinterpreted in this context, further to the ways we outlined earlier, and, where there may be a “bias … around mothers.” This participant reflected on how such concerns may be linked to a victim/survivor's hesitance to involve the police, especially if BWC footage will be captured:

All of the reasons why we’ve articulated women may not want police to attend with body-worn cameras, are the same reasons why they don’t want police to attend at all, because of the unintended consequences that may have on her safety … [and the] perception of how she's viewed as a parent.

Two participants described their experiences supporting victim/survivors who have either been at risk of losing or had lost, their children partly due to the misinterpretation of BWC footage. The first explained a situation in which the children supported the violent aggressor, which they suggested was linked to how children respond differently to violence:

[We] see women whose children will heavily align with the person who's using violence, and so whether that's Dad or Stepdad, they may align really heavily with that person and really want to be with that person; sometimes it's for safety reasons, sometimes because it's in their best interests to align with that person then they won’t be targeted. Sometimes we will see children be very withdrawn, they may be aggressive themselves, they may be very frightened and scared, and those responses from children can be misinterpreted. Those responses could be used against victims of domestic violence. (P18, administrative and family violence practitioner, QLD)

Another participant reflected on how the victim/survivor had her children removed and her concerns resulted in her being seen as “hysterical,” which created further problems for her in both gaining custody and going through the court system:

She was very emotionally distressed, and she had safety concerns for the children … she was very worried for her children's safety. … She was very emotionally heightened and the police response did not assist in de-escalating that … or validating her safety concerns … but [instead], reinforced that the system was supportive of her ex-partner who had committed horrific violence over a 13 year relationship. (P18, administrative and family violence practitioner, QLD)

This points both to unintended consequences of BWCs and the need to contextualize recordings of BWCs to recognize the trauma victim/survivors experience and how this may manifest in footage. Failure to understand the effects of DFV and think critically about the heavy—potentially impossible—expectations of (“ideal”) victim/survivors, means the footage can be used to harm, punish, or even criminalize, as opposed to assisting women and their families.

## Conclusion

Our findings suggest that BWC footage has the potential to show DFV and the impact of (primarily) physical harm. It may even encourage police and judicial officers “to look at things differently or in a different context” (P12, high-risk systems advocate, integrated response, QLD). However, concerns prevailed among participants that BWCs do not strictly replicate what victim/survivors witness or experience, particularly because they cannot capture the complete context and pattern of DFV, including nonphysical harms, such as controlling behaviors.

Additionally, most participants upheld the view that “a police officer's … bias, whether or not they have a camera” can impact how they respond to an incident, as well as how BWC footage may be viewed and interpreted (P12, high-risk systems advocate, integrated response, QLD). Differences in how police and judicial officers view and perceive BWC footage are also inherently connected to individual ideological, cultural, political, and social beliefs, as these factors guide cognitive biases, mental frameworks, and identity. This means that no two people will view BWC footage in the same way, which might result in differential applications and outcomes for some victim/survivors (see also Harris, 2020). The impacts of this can be incriminating for victim/survivors and may even result in the removal of children, especially for women who do not conform to “ideal victim” constructs or present as “expected” in BWC evidence to an untrained DFV expert.

This underscores the need for not only ensuring, but also advancing, trauma-informed responses among criminal justice practitioners to avoid adverse consequences for victim/survivors, such as misidentification or child removal. BWCs can and should be used as a tool in training criminal justice practitioners to better understand and respond to DFV, and enhance sensitive, trauma-informed responses to victim/survivors, and this should be achieved with the guidance of the DFV support sector, and specialist women's and legal services, who provide crisis responses and support to victim/survivors. Additionally, our findings show that there is merit in the use of BWC footage in court, as it has the potential to show a powerful account of a scene and the real-time contexts, including proceeding impacts, of abuse. In this way, the presentation of BWC footage in court may even serve as an educative tool for police, the community, and victim/survivors. Although, we would urge caution around who is responsible for BWC interpretation, and indeed feel that specialized DFV service providers ought to be involved in the analysis of BWC footage.

Additionally, while technical and practical constraints associated with “length, clarity, lighting, distance, angle, scope, steadiness, manner of shooting, [and] quality” (Wasserman, 2015, p. 840), including the speed and amount of movement at a scene, were not addressed in this article, these factors will inform pivotal considerations in forthcoming work given they might also impact how BWC footage is interpreted.

As with all studies, there are some limitations to this research. This paper is based on a small sample of 30 participants, with an uneven distribution between WA (*n *= 6) and QLD (*n *= 24) and does not offer generalizable findings, though it does offer opportunities to gain deep insight into the research topic. At the time of undertaking fieldwork, BWCs had only recently been introduced to WA (in 2016 trials, before being rolled out in 2019–2020), in comparison to QLD where they were introduced in 2015. As such, during recruitment, we found that more DFV stakeholders in QLD had knowledge of BWCs and experience with supporting victim/survivors who had encountered them and were therefore more willing and able to speak to us than DFV stakeholders in WA. We note that the sector is overburdened and under-resourced, so recruitment is challenging.

We recognize the benefits of mixed methods research and indeed use this in other components of our project, when assessing police perceptions of BWCs (see, Iliadis et al., 2022). Critiques of solely qualitative research abound, but we contend that the methods selected (semi-structured interviews) provide an in-depth reflection and understanding of the context in which BWCs record and footage are viewed; constructs of victimhood; manifestations of violence and trauma; and the differential impacts and effects on marginalized groups which has been missed in much research. This is, we believe, a consequence of the cohorts engaged in the literature, which has typically not included victim/survivors or advocates and practitioners other than police.

There is a difference between how BWCs operate in practice and in theory, and how success is understood by different cohorts. Unfortunately, victim/survivors are often overlooked. We recognize that victim/survivor voices were not included here, though it has been the focus of our other work and broader research agenda (Harris, 2020). We stress that it is imperative that future research and reviews of BWCs not only engage but prioritize victim/survivor voices.

Ultimately, the deployment of BWCs in DFV responses has been promoted as having the potential to transform police responses to victim/survivors’ and alleviate the burden, discomfort, and danger associated with State responses. As demonstrated throughout this article, however, debate abounds as to whether, and to what extent, BWCs can achieve these outcomes. For these reasons, we conclude that while BWCs have the potential to assist victim/survivors in formal responses, their true merits can only be reached with adequate police and judicial training that is trauma-informed and guided by the DFV support sector. This is important not only to mitigate the risk of immediate and ongoing violence, but also to ensure that BWCs are positioned within a consortia of trauma-informed State responses to DFV to promote victim/survivors’ safety and well-being.

## Notes

1. As outlined above, at the time of undertaking fieldwork, BWCs had only recently been introduced to WA (in 2016 trials, before being rolled out in 2019–2020), in comparison to QLD where they were introduced in 2015. We therefore found that during recruitment, more DFV stakeholders in QLD had supported victim/survivors who had encountered BWCs, and were thus better positioned to speak to us than DFV stakeholders in WA.
